# Characterization of Odor-Active 2-Ethyldimethyl-1,3,6-trioxocane Isomers in Polyurethane Materials

**DOI:** 10.3390/polym16243573

**Published:** 2024-12-21

**Authors:** Charlotte Minig, Alexandra Meißner, Martin Steinhaus

**Affiliations:** 1 Department of Chemistry, TUM School of Natural Sciences, Technical University of Munich, Lichtenbergstraße 4, 85748 Garching, Germany; charlotte.minig@tum.de; 2 Leibniz Institute for Food Systems Biology at the Technical University of Munich, Lise-Meitner-Straße 34, 85354 Freising, Germany; a.meissner.leibniz-lsb@tum.de

**Keywords:** polyurethane, polyether polyol, odorant, trioxocane, gas chromatography-olfactometry

## Abstract

Polyurethane materials, widely used in indoor environments, occasionally exhibit unpleasant odors. An important source of polyurethane odorants is polyether polyols. Previous studies identified odorous 2-ethyldimethyl-1,3,6-trioxocanes in polyurethane materials and polyols but did not investigate the odor activity of the individual isomers. In the present work, an isomer mixture of the precursor dipropylene glycol was fractionated through preparative high-performance liquid chromatography. After the conversion to the corresponding trioxocanes, gas chromatography-olfactometry analyses revealed that just one positional isomer, namely 2-ethyl-4,7-dimethyl-1,3,6-trioxocane, was odor active. Moreover, we observed clear differences in the odor threshold concentrations among its stereoisomers. Only two out of eight isomers displayed an odor, both with an earthy smell and one being approximately 60 times more potent than the other. These insights contribute to a better understanding of polyurethane odor on a molecular level and provide a basis for effective odor control.

## 1. Introduction

Polyurethanes (PUs) are among the most abundant and versatile plastic materials [1]. Most often, they are prepared from polyisocyanates and polyols. Polyols employed in PU production generally are polymeric compounds with 2–8 hydroxy groups and a maximum molecular weight of 10,000 g/mol [2]. Polyols can be classified by their polymeric backbone into, for example, polyether, polyester, and polycarbonate polyols [3]. Among these, polyether polyols obtained by polymerization of ethylene oxide and propylene oxide are most commonly used in PU production [4,5]. They provide flexible segments to the PU material and are appreciated for their low price and high hydrolytic stability [3].

Flexible PU foams make up the majority of PU materials [3,6]. They primarily serve as cushioning materials in automotive vehicles, mattresses, and upholstered furniture [3]. Thus, they are highly abundant in indoor environments, and their odor has to be carefully controlled. An obtrusive odor may lead to the rejection of an otherwise acceptable PU material, causing avoidable economic loss and ecological damage. Any raw material in PU production could, in principle, introduce unwanted odorants, but polyether polyols stand out as they are both widely used and a main ingredient in most PU formulations. Polyol weight percentages are typically around 30% in rigid PU foams used for insulation and as high as 70% in flexible PU foams [4]. Therefore, they represent a promising target for PU odor reduction efforts.

So far, two studies have identified polyether polyols as a potential source of odorous trace compounds in PUs. Harris et al. were the first to describe “musty”-smelling compounds when applying activity-guided odorant screening to PU foam and polyol solvent extracts [7]. To test the hypothesis that these were cyclic species consisting of propylene oxide units, they treated propylene oxide with the Lewis acid boron trifluoride. The product was purified by fractional distillation and preparative gas chromatography. Finally, using spectrometric methods, the odorants were structurally identified as 2-ethyldimethyl-1,3,6-trioxocanes, that is, cyclic acetals of dipropylene glycol (DPG) and propanal. Harris et al. proposed their acid-catalyzed formation from propylene oxide during polyol production. This mechanism allows the formation of three positional isomers and as many as fourteen different stereoisomers, but the authors did not attempt to further characterize the structures of the odorants.

Recently, we reported detecting this type of odorant in various PU materials and a polyether polyol [8]. Volatile isolates of the samples, obtained via solvent extraction in combination with automated solvent-assisted flavor evaporation [9], were subjected to gas chromatography-olfactometry (GC-O). Odorants were then identified by comparing their odor quality, retention behavior, and mass spectrum to those of authentic reference compounds. Based on the results reported by Harris et al. [7], reference trioxocanes were synthesized from propanal and technical grade DPG. The latter is a mixture of positional and stereoisomers formed as by-products in the industrial production of propylene glycol from propylene oxide [10]. As expected, this synthetic approach resulted in a mixture of trioxocane isomers. However, GC-O analysis of the mixture revealed that only a single peak exhibited the strong earthy odor perceived in the PU materials, whereas the other peaks showed little or no odor at all. From the available data, it was unclear which of the fourteen possible 2-ethyldimethyl-1,3,6-trioxocane isomers was the potent odorant. To draw near to the structure of this earthy-smelling compound, we fractionated a commercial DPG mixture by preparative high-performance liquid chromatography (HPLC), fraction-wise reacted the separated DPG isomers with propanal, and analyzed the resulting trioxocanes by achiral and chiral GC-O.

## 2. Materials and Methods

### 2.1. Chemicals

Propanal (≥99%) and Amberlite IR-120H were from Thermo Fisher Scientific (Dreieich, Germany). Technical grade DPG (99%), anhydrous sodium sulfate, (2*E*)-dec-2-enal (≥95%), and pentane-1,5-diol (96%) were from Merck (Darmstadt, Germany). 2,2′-Oxydi(propan-1-ol) (99%) and 2,4,6-triethyl-1,3,5-trioxane (95%) were from Enamine (Riga, Latvia). Ethanol (≥99.8%) and *n*-hexane (≥95%) were from Honeywell (Offenbach am Main, Germany). Dichloromethane and *n*-pentane were from CLN (Freising, Germany) and were freshly distilled before use.

### 2.2. Gas Chromatography–Olfactometry (GC-O)

The GC-O system consisted of a Trace GC Ultra gas chromatograph (Thermo Fisher Scientific) equipped with a cold on-column injector, a flame ionization detector (FID), and a custom-made sniffing port [11]. GC-O analyses were performed with an achiral FFAP column (30 m × 0.32 mm i.d., 0.25 µm film thickness) purchased from Agilent (Waldbronn, Germany) or a chiral BGB-176 column (30 m × 0.25 mm i.d., 0.25 µm film thickness) purchased from BGB Analytik (Rheinfelden, Germany), whose stationary phase was 30% 2,3-dimethyl-6-*tert*-butyldimethylsilyl-β-cyclodextrin dissolved in BGB-15 (15% phenyl-, 85% methylpolysiloxane). The carrier gas was helium at a constant pressure of 70 kPa (FFAP) or 100 kPa (BGB-176). The injection volume was 1 µL. When the FFAP column was used, the initial oven temperature of 40 °C was held for 2 min, then the temperature was raised at a rate of 6 °C/min to a final temperature of 230 °C, which was held for 5 min. With the BGB-176 column, the initial temperature of 40 °C was held for 2 min, and then the temperature was raised at a rate of 1 °C/min to 130 °C. Afterward, the temperature increased by 6 °C/min to the final temperature of 200 °C, which was held for 5 min. The column effluent was split into two equal parts using a deactivated Y-shaped glass splitter and two deactivated fused silica capillaries (50 cm × 0.25 mm i.d.). One half was directed to the FID (base temperature 250 °C) and one half to the sniffing port (base temperature 250 °C). During a run, an assessor placed the nose directly above the sniffing port and noted any perceived odor impressions. Data were evaluated using the ChromQuest 5.0 software (Thermo Fisher Scientific).

### 2.3. Gas Chromatography–Flame Ionization Detector (GC-FID)

The GC-FID system consisted of the components detailed in Section 2.2, but without a sniffing port. Injection was carried out with a Triplus autosampler (Thermo Fisher Scientific).

### 2.4. Gas Chromatography–Mass Spectrometry (GC-MS)

The GC-MS system consisted of a Trace GC Ultra gas chromatograph coupled to a single quadrupole ISQ mass spectrometer (Thermo Fisher Scientific), equipped with a GC PAL autosampler (CTC Analytics; Zwingen, Switzerland) and a programmed temperature vaporizing injector. During injection, the temperature was raised from the starting temperature (40 °C) with 12° C/s to 60 °C (held for 30 s) and then with 10 °C/s to 240 °C (held for 60 s). The injector was used in split mode with a split flow of 24 mL/min. GC-MS analyses were performed with an FFAP column (30 m × 0.25 mm i.d., 0.25 µm film thickness) purchased from Agilent. The carrier gas was helium at a constant flow of 1.2 mL/min. The injection volume was 1 µL. The temperature program described in Section 2.3 was used. Mass spectra were generated in electron ionization mode at 70 eV and with a scan range of 40–300 *m*/*z*. Data were evaluated using the Xcalibur 4.0 software (Thermo Fisher Scientific).

### 2.5. Fractionation of Dipropylene Glycol Isomers by Preparative High-Performance Liquid Chromatography (HPLC)

The HPLC system (Jasco; Pfungstadt, Germany) consisted of an autosampler AS-2057 Plus, a gradient mixer LG-1580-02, a pump PU-1580, and a refractive index detector RI-2031 Plus. Preparative separation of the DPG isomers was carried out with a Eurospher II 100-5 diol column, 250 × 8 mm, together with a Eurospher II 100-5 diol precolumn, 5 × 4 mm (Knauer; Berlin, Germany). The injection solution consisted of technical-grade DPG dissolved (20 mg/mL) in *n*-hexane/ethanol (75 + 25, v + v). The injection volume was 50 µL. Isocratic elution was performed with *n*-hexane/ethanol (90 + 10, v + v) at 1.6 mL/min. The chromatography was monitored using the Galaxie 1.10 software (Agilent).

Through portion-wise GC-FID analysis of the eluate, three distinct regions in the chromatogram were identified where specific DPG isomers eluted. In the following runs, the eluate during these periods was collected manually. All fractions from 56 runs containing the same DPG isomer(s) were combined. The solvent was removed by rotary evaporation (50 mbar), and the residues were dissolved in dichloromethane/*n*-pentane (5 + 1, v + v; 15 mL). The DPG concentration in the resulting solutions was determined in duplicates by GC-FID. Pentane-1,5-diol was used as the internal standard with a response factor determined by analyzing mixtures with known concentrations of DPG and pentane-1,5-diol.

### 2.6. Synthesis of 2-Ethyldimethyl-1,3,6-trioxocanes

For the synthesis of a reference mixture of 2-ethyldimethyl-1,3,6-trioxocane isomers, technical grade DPG (3.0 g, 22 mmol) and propanal (freshly distilled over sulfuric acid; 1.8 g, 31 mmol) were combined with a strongly acidic cation exchange resin (Amberlite IR-120H; 0.71 g), anhydrous sodium sulfate (~10 g), and a dichloromethane/*n*-pentane mixture (5 + 1, v + v; 100 mL) in a round-bottom flask [8]. The mixture was refluxed for 2 h. Solids were removed by filtration. The filtrate was washed with aqueous sodium carbonate solution (0.25 mol/L; 2 × 50 mL), dried over anhydrous sodium sulfate, and made up to 100 mL.

For the synthesis of trioxocanes from the purified DPG isomeric mixtures obtained by HPLC fractionation, a miniaturized version of the above protocol was used. The diols (6.2–7.1 mg, 46–53 µmol) were combined with the ~1.4-fold stoichiometric amount of freshly distilled propanal, ion exchanger (1.9–2.3 mg), and anhydrous sodium sulfate (~1.0 g) in a glass test tube (30 mL) with a screw cap [12]. The mixture was stirred at 55 °C for 2 h and then filtered. The filtrate was washed with aqueous sodium carbonate solution (0.25 mol/L; 2 × 20 mL), dried over anhydrous sodium sulfate, and concentrated to 1 mL using first a rotary evaporator and then a Bemelmans microdistillation device [13]. Trioxocanes in the mixture were quantitated in duplicates using the GC-FID instrument with 2,4,6-triethyl-1,3,5-trioxane as the internal standard.

### 2.7. Determination of Odor Threshold Concentrations (OTCs) in Air

OTCs were determined by GC-O analysis of successively 1:2 diluted mixtures containing the target odorants and the internal standard (2*E*)-dec-2-enal [14]. Analyses were performed using the BGB-176 column by a total of three assessors. The OTCs were calculated from the dilution factors, the concentrations of the standard and the analytes, and an OTC of 2.7 ng/L air for (2*E*)-dec-2-enal [15].

## 3. Results and Discussion

### 3.1. Characterization of Trioxocanes in a Reference Mixture

**Figure 1:**
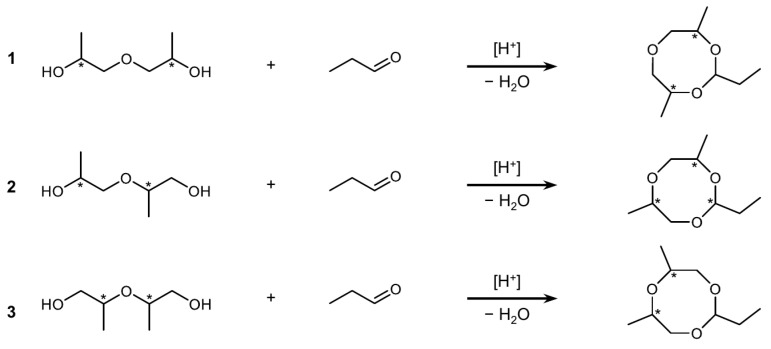
Synthesis of 2-ethyldimethyl-1,3,6-trioxocanes from isomeric dipropylene glycols (**1**–**3**) and propanal. Asterisks indicate the positions of the stereogenic centers.

As a first step toward identifying the PU odor-causing trioxocane isomers, we looked more closely at the odor activity of the individual components in a trioxocane reference mixture. We synthesized this mixture by reacting technical-grade DPG, which contains 1,1′-oxydi(propan-2-ol) (**1**), 2-(2-hydroxypropoxy)propan-1-ol (**2**), and 2,2′-oxydi(propan-1-ol) (**3**), with propanal in the presence of a solid acid catalyst (Figure 1) [12,16].

**Figure 2:**
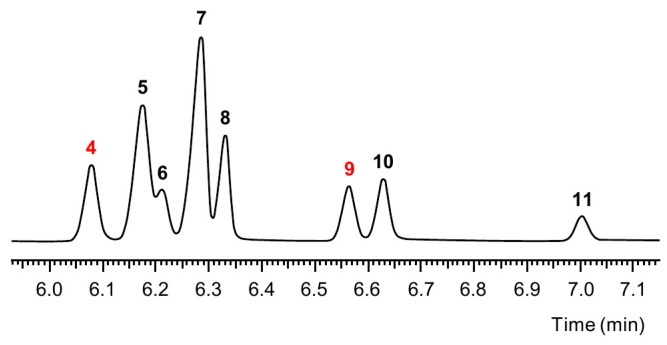
GC-MS chromatogram (FFAP column) of a mixture of 2-ethyldimethyl-1,3,6-trioxocane isomers. Red numbers indicate odorous compounds.

The mixture was analyzed by means of GC-MS using an FFAP column (Figure 2). Apart from residual DPG, it contained eight closely eluting compounds (**4**–**11**) whose mass spectra were compatible with the expected 2-ethyldimethyl-1,3,6-trioxocane structures. Given that 2-ethyl-4,8-dimethyl-1,3,6-trioxocane and 2-ethyl-5,7-dimethyl-1,3,6-trioxocane, derived from **1** and **3**, respectively, possess a plane of symmetry and only two stereogenic centers, their stereoisomers consist of one enantiomeric pair and one achiral *meso* isomer each. Thus, these positional isomers explained four of the eight peaks. The remaining four peaks could be attributed to the four enantiomeric pairs of 2-ethyl-4,7-dimethyl-1,3,6-trioxocane, an asymmetric compound with three chirality centers.

Before investigating which peak corresponded to which isomer, we first assessed the relative odor activity of the substances behind the eight peaks. For that purpose, the trioxocane reference mixture was stepwise diluted 1:10, and the diluted solutions were subjected to GC-O. The relative odor activities of **4**–**11** were approximated from the respective dilution factors, corresponding to the highest diluted solution in which an odor was perceptible, and the area count percentages of the compounds in the reference mixture followed by normalization. The results (Table 1) indicated that **9** was the most potent odorant among the trioxocanes, thus being assigned a relative odor activity of 1.00. The second most potent odorant was **4**, but with a clearly lower relative odor activity of only 0.07. All other isomers showed relative odor activities <0.02 and were, therefore, not considered relevant for PU odor problems. In agreement with these findings, our earlier study identified **9** (retention index 1409) as an odorant in the volatile fractions of most of the PU material samples [8]. By contrast, **4** (retention index 1366) was detected by GC-O only in the volatiles isolate of a polyether polyol but not in the isolates of the PU samples. The other trioxocane isomers, though detectable in the GC-MS chromatograms, were not perceived in the sensory-directed activity-guided screening. These results prompted us to put a particular focus on the structure elucidation of compounds **4** and **9**.

### 3.2. Annotation of Dipropylene Glycol Isomers

**Figure 3:**
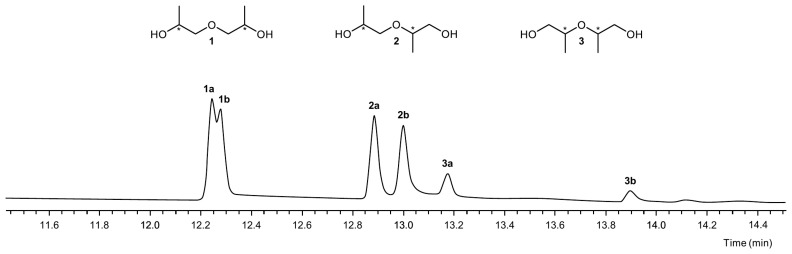
GC-FID chromatogram (FFAP column) of technical grade DPG. Asterisks indicate the positions of the stereogenic centers.

To assign the trioxocane peaks to the three positional isomers, we aimed to isolate the precursor compounds **1**–**3** from the technical-grade DPG mixture and to separately react them with propanal. GC analysis of the technical grade mixture resulted in six peaks, the first two eluting very closely together (Figure 3). The separation was not substantially improved by using different stationary phases, including polyethylene glycol-based ones, a longer column (50 m), a higher film thickness (1.2 µm), a capillary with a smaller inner diameter (0.25 mm), or a lower oven temperature rate (2–4 °C/min).

**Figure 4:**
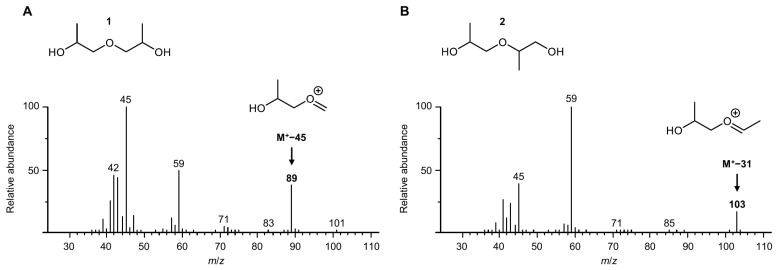
Electron ionization mass spectra of (**A**) 1,1′-oxydi(propan-2-ol) and (**B**) 2-(2-hydroxypropoxy)propan-1-ol.

The fifth and sixth peaks could be annotated to 2,2′-oxydi(propan-1-ol) (**3**) by comparison with a purchased reference standard. For the annotation of the remaining signals, the electron ionization mass spectra were evaluated. A search in the NIST mass spectral database [17] indicated that the first two peaks corresponded to 1,1′-oxydi(propan-2-ol) (**1**). The fragment ion *m*/*z* 103 (M^+^−31) was virtually absent from their mass spectra (Figure 4A). However, there was a pronounced signal with *m*/*z* 89 (M^+^−45). The spectra of any of the other peaks closely resembled the reference spectra of 2-(2-hydroxypropoxy)propan-1-ol (**2**) and 2,2′-oxydi(propan-1-ol) (**3**). In these spectra (Figure 4B), *m*/*z* 103 was clearly visible, whereas the intensity of *m*/*z* 89 was very low. Both ions could be explained by an α-fragmentation [18], but apparently, when the molecular ion contained only secondary hydroxyl groups, a C_2_H_5_O radical (45 u) was cleaved. By contrast, a primary hydroxyl function predominantly resulted in the loss of a CH_3_O radical (31 u). Therefore, the stereoisomers of **1**, the only ones without a terminal hydroxyl function, were identified as the compounds responsible for the first two peaks. The elution order we determined was in line with the results reported by Schaefer et al. [19], who separated technical grade DPG using a packed mercury chloride/carbowax column. They confirmed the assignment by nuclear magnetic resonance spectroscopy after the derivatization of the diols with trichloroacetyl isocyanate. Moreover, primary *n*-alkanols generally have higher RI values than their secondary positional isomers [20], and the peak area ratios were in agreement with previous literature reporting that **3** was the minor positional isomer in the technical grade mixture [19,21].

**Table 1:** Retention indices and relative odor activities of trioxocanes synthesized from technical grade DPG.

Compound	Retention Index ^1^	Relative Odor Activity ^2^
**4**	1366	0.07
**5**	1372	<0.01
**6**	1374	<0.01
**7**	1381	<0.01
**8**	1385	<0.01
**9**	1409	1.00
**10**	1414	<0.01
**11**	1448	<0.02

^1^ Calculated by linear interpolation from the retention time of the compound and the retention times of adjacent n-alkanes on the FFAP column [22]. ^2^ Calculated from the dilution factor of the highest diluted solution obtained by serial 1:10 dilutions of the trioxocane reference mixture in which the odorant was detected during GC-O analysis, divided by the FID area count percentage of the compound in the mixture, followed by normalization.

### 3.3. Characterization of Individual Trioxocane Isomers

**Figure 5:**
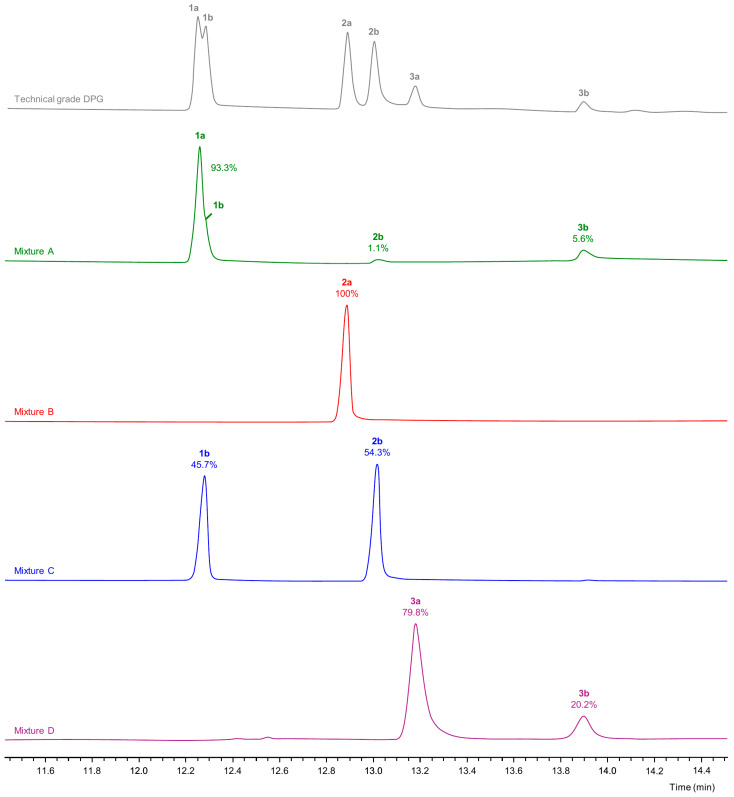
GC-FID chromatograms (FFAP column) of mixtures obtained via liquid-chromatographic separation of technical grade DPG. Numbers in bold refer to the DPG isomers 1,1′-oxydi(propan-2-ol) (**1**), 2-(2-hydroxypropoxy)propan-1-ol (**2**), and 2,2′-oxydi(propan-1-ol) (**3**).

Preparative separation of the DPG isomers was carried out by HPLC. Since ultraviolet detection was not feasible due to a lack of chromophores within the molecule, a refractive index detector was employed. DPG isomers were recovered in a total of three fractions (Figure 5, mixtures A–C). Mixture A consisted mainly of **1a**. Additionally, it contained minor amounts of **1b**, **2b**, and **3b**. In mixture B, only **2a** was detected, and mixture C contained **1b** and **2b**. Mixture D was the commercially obtained 2,2′-oxydi(propan-1-ol) and consisted of **3a** and **3b** in a ratio of ~4:1.

**Figure 6:**
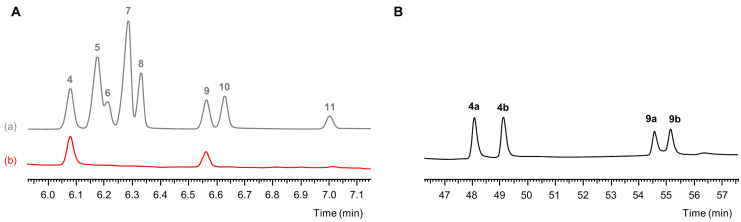
(**A**) GC-FID chromatograms (achiral FFAP column) of trioxocanes synthesized from (a) technical grade DPG and (b) DPG isomer **2a**. (**B**) GC-FID chromatogram (chiral BGB-176 column) of 2-ethyl-4,7-dimethyl-1,3,6-trioxocane isomers.

After the synthetic protocol had been applied to DPG mixtures A–D, the resulting trioxocanes were annotated to compounds **4**–**11** (Table 2) based on their retention indices [22]. When applying achiral GC-O, no odorous regions were detected in the trioxocane mixtures obtained from DPG mixtures A, C, and D. On the other hand, the trioxocane mixture obtained from DPG mixture B showed two odorous peaks, which matched the odor-active compounds **4** and **9** in the trioxocane reference mixture obtained from the technical grade DPG (Figure 6A). Since DPG mixture B had contained only one enantiomeric pair (**2a**) of the asymmetric DPG isomer 2-(2-hydroxypropoxy)propan-1-ol, the sniffing results suggested that only 2-ethyl-4,7-dimethyl-1,3,6-trioxocanes are relevant as PU odorants, whereas 2-ethyl-4,8-dimethyl-1,3,6-trioxocanes and 2-ethyl-5,7-dimethyl-1,3,6-trioxocanes are not.

The trioxocane mixture obtained from DPG mixture B was further analyzed using the chiral column. The chromatogram displayed four peaks (Figure 6B), representing the enantiomeric pairs of **4** and **9**, respectively. The peak areas indicated the formation of racemic mixtures of **4a** and **4b** as well as **9a** and **9b**. Finally, GC-O in combination with an internal reference compound was used to estimate their absolute odor threshold concentrations in air (Table 3). No odor activity was observed for stereoisomers **4a** and **9b**. For trioxocane **9a**, we determined an OTC over 60 times lower than that of **4b**. The GC-O analyses of the stepwise diluted trioxocane reference mixture (cf. Section 3.1) had suggested an OTC of the enantiomeric pair **9** approximately one order of magnitude lower than that of pair **4**. Hence, their relative odor activities and their OTCs aligned well. It should be noted that the OTC determination provided a more accurate evaluation of odor activity because the dilution steps were smaller (1:2 vs. 1:10) and the number of assessors higher (3 vs. 1).

Considerable differences in odor potency, as seen in the trioxocanes, are frequently observed between stereoisomers. This principle is evident, among others, in the wine lactone 3a,4,5,7a-tetrahydro-3,6-dimethylbenzofuran-2(3*H*)-one. The OTCs of its eight stereoisomers range from >1000 to 0.00001 ng/L air, with the (3*S*,3a*S*,7a*R*)-enantiomer exhibiting an OTC 700 times lower than that of the second most potent stereoisomer [23]. Further examples include (*R*)-linalool and (*S*)-linalool (OTCs in water: 0.82 µg/kg vs. 8.3 µg/kg) [24], and (*R*)-δ-2-decenolactone and (*S*)-δ-2-decenolactone (OTCs in air: 1.6 ng/L vs. 52 ng/L) [25].

## 4. Conclusions

Cyclic acetals of dipropylene glycol and propanal have been reported as odorants exclusively in flexible PU and its raw materials so far. The current study revealed that one enantiomer of 2-ethyl-4,7-dimethyl-1,3,6-trioxocane is a far more potent odorant than any of the thirteen other 2-ethyldimethyl-1,3,6-trioxocane isomers. Consequently, our results strongly suggest this structural isomer as the primary cause of the earthy odor that trioxocanes impart to PU materials. Further research should be conducted on its formation kinetics to clarify how its generation can be prevented.

Differentiating highly similar trace compounds, such as trioxocane isomers, poses a considerable analytical challenge. Nevertheless, we demonstrated that their gas chromatographic separation is possible down to the level of enantiomers. However, since the odorous enantiomers and their odorless counterparts are formed in virtually equal amounts, we consider achiral GC sufficient for routine analytical purposes. Monitoring odorous trioxocanes in the quality control of polyols and PU materials may enable manufacturers to predict odor outcomes based on molecular data, rather than relying solely on sensory testing of the finished materials.

## Figures and Tables

**Table 2:** Main trioxocanes obtained from DPG mixtures A–D.

Mixture	Main DPG Isomer (s)	Main Trioxocane (s)
A	**1a**	**8**
B	**2a**	**4**, **9**
C	**1b**, **2b**	**5**, **7**, **10**
D	**3a**, **3b**	**6**, **11**

**Table 3:** Odor threshold concentrations of 2-ethyl-4,7-dimethyl-1,3,6-trioxocane stereoisomers.

Stereoisomer	Odor Threshold Concentration ^1^ (ng/L Air)
**4a**	>1.4
**4b**	1.2
**9a**	0.019
**9b**	>0.8

^1^ Determined as described by Ullrich and Grosch [14].

## Data Availability

The original contributions presented in this study are included in the article. Further inquiries can be directed to the corresponding author.
